# Neonatal outcomes in women with preterm premature rupture of membranes at periviable gestational age

**DOI:** 10.1038/s41598-022-16265-5

**Published:** 2022-07-14

**Authors:** Jacky Herzlich, Laurence Mangel, Ariel Halperin, Daniel Lubin, Ronella Marom

**Affiliations:** 1 Department of Neonatology, Dana Dwek Children’s Hospital, Tel Aviv Sourasky Medical Center, Tel Aviv, Israel; 2 Department of Obstetrics, Gynecology, Mayanei HaYeshua Center, Bnei Brak, Israel; 3 Department of Neonatology, Mayanei HaYeshua Center, Bnei Brak, Israel; 4 Sackler Faculty of Medicine, Tel Aviv University, Tel Aviv, Israel; 5 Department of Neonatology, Dana Dwek Children’s Hospital, Tel Aviv Sourasky Medical Center, 6 Weizmann St, 6423906 Tel Aviv, Israel

**Keywords:** Health care, Medical research

## Abstract

To examine the outcomes of preterm infants born to women with preterm premature rupture of membranes (PPROM) at periviable gestational age. This is an observational retrospective cohort study analyzing data collected on singleton deliveries complicated by prolonged premature rupture of membranes occurring between 17 and 33 weeks of gestation. Neonatal outcomes including birth weight, Apgar score, retinopathy of prematurity, intraventricular hemorrhage, bronchopulmonary dysplasia, necrotizing enterocolitis, hearing impairment and mortality were evaluated. Ninety-four preterm infants who were born after a prolonged premature rupture of membranes of at least 7 days were included in the study. Median gestational week at onset of membrane rupture was 27.1 ± 4.2 weeks (range 17–33) and median latency period in days was 16 ± 21.8 (range 7–105). The cohort was stratified by gestational week (GW) at onset of PPROM (group 1: 17–23, group 2: 24–27, and group 3: 28–33). We found that the survival rate to discharge within neonates born after prolonged rupture of membrane at gestational week less than 24 weeks is 79.2% and 88.9% in group 2. These neonates did not show an increased rate of major morbidities compared to neonates born following membrane rupture at gestational week 24 to 27. We described a high survival rate to discharge without major morbidities following prolonged preterm membrane rupture of at least 7 days of latency before viability.

## Introduction

Preterm premature rupture of membranes (PPROM) defined, as the spontaneous rupture of membranes before 37 weeks of gestation, is a relatively common complication in pregnancy. It occurs in about 5 to 7% of pregnancies^1^ and accounts for one-third of preterm births^2,3^. The incidence of PPROM, at periviable gestational age (broadly defined as 20 0/7 through 25 6/7 weeks of gestation)^4^ is less common, affecting approximately four in 1000 pregnancies^5^. PPROM exposes the fetus to infection, abruptio placentae, cord prolapse, and intrauterine death, during the latency period (time between onset of PPROM to birth)^5^. The neonatal outcome is generally poor as a result of preterm delivery, inflammatory response, and a certain degree of pulmonary hypoplasia stemming from the reduction in amniotic fluid at a very early gestational age (GA)^6,7^. The optimal management of pregnancies complicated by PPROM remains a challenge. The issue of expectant management vs. immediate delivery, especially in cases of PPROM occurring at less than 30 gestational week (GW), is controversial^6–11^. The last Cochrane on this issue recommended a policy of expectant management between 25 and 37 weeks of pregnancy with careful monitoring to achieve better outcomes for the mother and her baby^12^.

The current approach in Israel to pregnancies presenting with PPROM falls into three categories as a function of GW^13^. Before 23 weeks, the recommendation is to terminate the pregnancy; between 23 + 0 and 23 + 6 weeks, the decision on pregnancy termination is made following consultation between the medical team and the parents; and above 24 weeks, expectant management, including corticosteroids and antibiotics administration, is the general rule.

Although these gestational age-based guidelines are essential to health care providers in the maternal/neonatal treatment decision-making, counselling parents on the rate of neonatal survival and long-term disabilities is a challenging complex issue, and present women with dilemmas in view of individual circumstances and patient values.

The primary aim of this study was to describe the outcomes, especially survival rate, of preterm infants born following management of prolonged PPROM occurring from 17 to 33 weeks with PROM of more than 7 days.

## Materials and methods

This is a retrospective analysis of data on singleton deliveries complicated by prolonged PPROM (≥ 1 week) occurring between 17 and 33 weeks of gestation, conducted at the Mayanei HaYeshua Medical Center (MHMC) between January 2014 and December 2019. The local ethics committee of the Mayanei Hayeshua Medical Center approved the present study (0015-19-MHMC) and waived the need for informed consent, due to the retrospective character of the study. The study was carried out in accordance with Good Clinical Practice guidelines and the Declaration of Helsinki. We included preterm babies born following prolonged PPROM and admitted to the Neonatal Intensive Care Unit (NICU). Data were extracted from medical records, included were maternal data on age, medical history and comorbidities (e.g. gravidity, parity, previous preterm delivery, hypothyroidism, diabetes mellitus, gestational diabetes mellitus, hypertension, epilepsy, thrombophilia, the use of selective serotonin reuptake inhibitors (SSRIs), group B streptococcus (GBS) colonization), chorioamnionitis, maternal fever, GW at PPROM onset, mode of delivery and duration of latency. Neonatal data on GA, birth weight (BW), gender, APGAR score at 1 and 5 min, intubation and days of ventilation, mode of ventilation or assisted ventilation, nitric oxide (NO) inhalation, morbidities such as retinopathy of prematurity (ROP), intraventricular hemorrhage (IVH), periventricular leukomalacia (PVL), bronchopulmonary dysplasia (BPD), necrotizing enterocolitis (NEC) stage 2–3, patent ductus arteriosus (PDA), blood stream infection (BSI), hearing impairment, pulmonary hypertension, pneumothorax and central line blood stream infection (CLABSI)), medication (surfactant, diuretics and steroid treatment), and survival at discharge were recorded.

PPROM was diagnosed using the AmniSure ROM Test (Qiagen Sciences LLC, Germantown, MD, USA) which is based on a report of watery leakage from the vagina, confirmed by sterile speculum examination and the observation of either fluid accumulation in the posterior vaginal fornix or direct leakage from the cervical canal with pressure from uterine fundus or upon coughing. Cases of PPROM latency period less than 7 days before birth and PPROM occurring after 35 + 0 gestational weeks were excluded.

### Statistical analysis

Categorical variables are reported as frequencies and percentages. Descriptive statistics (means and standard deviations) were calculated for demographic data. Normality was assessed by Shapiro–Wilk tests. Fisher’s exact tests or Chi square tests were used to compare categorical variables between groups as appropriate and followed by a post-hoc Bonferonni correction when appropriate. Multiple comparisons of continuous variables among groups were performed using the Kruskal–Wallis test followed by post-hoc Bonferonni pairwise comparison or the one-way analysis of variance (ANOVA) followed by post-hoc Tukey HSD test, when appropriate. The Mann–Whitney U test was applied to compare continuous variables when appropriate. A Spearman’s rank correlation coefficient was used to test for correlation between GW at onset of PPROM, GA at delivery, duration of latency period, Apgar scores and BW. Binary logistic regression analysis was conducted to investigate whether variables (GA at delivery, GW at onset of PPROM, BW, Apgar scores and latency period length) could predict neonatal survival to discharge.

IBM SPSS Statistics for Windows, version 25, was used for statistical data analysis and p-values < 0.05 were considered statistically significant.

## Results

Ninety-four cases of PPROM (≥ 7 days) deliveries were included in the study period. None of the mothers underwent a prior amniocentesis. They were all admitted at onset of PPROM and were kept under observation at the maternal high-risk pregnancy ward until delivery. Demographic and clinical characteristics of the mothers and preterm infants are presented in Table 1. Briefly, the average maternal age was 28.4 ± 5.4 and the average GA at delivery was 30.8 ± 3.1 weeks (range 23–34). Median GW at onset of PPROM was 27.1 ± 4.2 weeks (range 17–33) and median length of latency period in days was 16 ± 21.8 (7–105). Corticosteroids, magnesium and antibiotics were administrated to almost all the mothers (97.9–98.9%).

**Table 1:** Maternal and neonatal characteristics.

Characteristics	GW at PPROM				p value
	Total (17–33) N = 94	Group 1 (17–23) N = 24	Group 2 (24–27) N = 27	Group 3 (28–33) N = 43	
**Maternal characteristics**					
Maternal age (year)	28.4 ± 5.4 (20–41)	29 ± 5 (22–41)	28.2 ± 5 (21–41)	28.2 ± 5.8 (20–41)	0.401
Gravidity	4.8 ± 3.2 (1–16)	4.8 ± 2.8 (1–12)	4 ± 2.2 (1–9)	5.1 ± 3.8 (1–16)	0.826
Parity	3 ± 2.4 (0–12)	3 ± 2.2 (0–8)	2.5 ± 1.9 (0–7)	3 ± 2.8 (0–12)	0.493
Celeston (2 courses)	93 (98.9)	23 (95.8)	27 (100)	43 (100)	0.229
Magnesium treatment	92 (97.9)	22 (91.7)	27 (100)	43 (100)	0.051
Treatment with antibiotics (mercer protocol)	93 (98.9)	24 (100)	26 (96.3)	43 (100)	0.285
Chorioamnionitis	18 (19.1)	4 (16.7)	11 (40.7)	3 (7)	**0.002**
Previous preterm delivery*	12 (12.8)	7 (29.2)	0	5 (11.6)	**0.024**
Placenta abruption	22 (23.4)	9 (37.5)	7 (25.9)	6 (14)	0.086
GW at onset of PPROM (median, week)	27 ± 4.2	22 ± 1.6	26 ± 1.2	31 ± 1.4	** < 0.001**
Latency period (median, day)	16 ± 21.8 (7–105)	49 ± 28.1 (12–105)	17 ± 14.8 (7–61)	12 ± 5.7 (7–26)	** < 0.001**
Cesarean delivery	45 (47.9)	17 (70.8)	14 (51.9)	14 (32.6)	**0.01**
**Neonatal characteristics**					
GA at delivery (week)	30.8 ± 3.1 (23–34)	28.5 ± 3.3 (23–34)	29.3 ± 2.4 (25–34)	33 ± 1.3 (30–34)	** < 0.001**
Male gender	60 (63.8)	14 (58.3)	20 (74.1)	26 (60.5)	0.416
Birth weight (g)	1615.1 ± 544.4 (488–2945)	1270.6 ± 472.1 (488–2120)	1347.7 ± 410.3 (730–2270)	1975 ± 428.4 (1145–2945)	** < 0.001**
Apgar score 1 min	6.8 ± 2.5 (1–9)	5.1 ± 2.4 (1–9)	6.2 ± 2.8 (1–9)	8.1 ± 1.6 (2–9)	** < 0.001**
Apgar score 5 min	9.6 ± 1.8 (3–10)	7.6 ± 1.4 (4–10)	8.3 ± 1.9 (4–10)	9.4 ± 1.3 (3–10)	** < 0.001**
Survived to Hospital discharge	86 (91.5)	19 (79.2)	24 (88.9)	43 (100)	**0.012**

Data are expressed as mean/median ± standard deviation (range) or n (%).

*GA* gestational age, *GW* gestational week.

*2 missing values.

Significant values are in bold.

**Figure 1:**
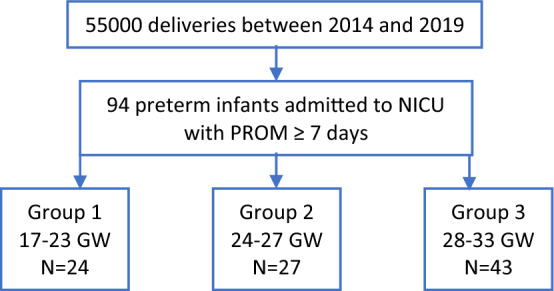
Flow chart of the cohort.

The cohort was further stratified by GW at onset of PPROM, as group 1 (17–23 weeks), group 2 (24–27) and group 3 (28–33) (Fig. 1, Table 1). There were no significant differences in maternal comorbidities including hypothyroidism, diabetes mellitus, gestational diabetes mellitus, hypertension, epilepsy, thrombophilia, maternal fever, group B streptococcus (GBS) colonization and maternal use of selective serotonin reuptake inhibitors (SSRIs), between the groups. However, the rate of chorioamnionitis, per Chi square analysis, was significantly higher in-group 2 (p ≤ 0.001) and lower in-group 3 (p = 0.006) after Bonferonni correction. Furthermore, mothers in group 3 had a significant lower rate of cesarean delivery (32.6%, p = 0.006) and significantly more mothers in group 1 (29.2%) had a previous history of preterm delivery, (p = 0.004) than the ones in the other groups after Bonferonni correction. By Kruskal–Wallis test with post hoc comparison, the median length of latency period, in group 3, was significantly shorter than in group 2 and 1 (12 ± 5.7 vs 17 ± 14.8 and vs 49 ± 28.1, p < 0.001, respectively).

By one-way ANOVA test with post hoc analysis, GA and BW in group 3 were significantly higher than in group 2 and 1 (p < 0.001). Similarly, Apgar score at 1 and 5 min in group 3 were significantly higher than in group 1 and 2, (p < 0.001 and p < 0.05, respectively). We found that the survival rate to discharge in neonates born to prolonged PPROM-complicated pregnancy at GW < 24 is 79.2%, compared to 88.9% in group 2 (p < 0.451) and 100% in group 3 (p < 0.012). We further compared neonatal outcomes between group 1 and group 2, the next closest group in terms of GW at PPROM onset (Table 2). There were no significant differences between group 1 and 2 for most of the reported parameters with the exception of use of surfactant (70.8% vs 37%, respectively, p = 0.025, two-sided Fisher’s exact test) and NO inhalation treatments (25% vs 3.7%, respectively, p = 0.042, two-sided Fisher’s exact test). Also, mean Apgar at 5 min in group 1 was significantly lower than in group 2, (7.6 ± 1.4 vs 8.3 ± 1.9, p = 0.029).

**Table 2:** Neonatal outcomes by gestational week (GW) at PPROM.

	GW at PPROM		p value
	Group 1, N = 24 (17–23)	Group 2, N = 27 (24–27)	
GA at delivery (week)	28.5 ± 3.3 (23–34)	29.3 ± 2.4 (25–34)	0.366^c^
Birth weight (g)	1270.6 ± 472.1 (488–2120)	1347.7 ± 410.3 (730–2270)	0.536^b^
Apgar 1	5.1 ± 2.4 (1–9)	6.2 ± 2.8 (1–9)	0.111^c^
Apgar 5	7.6 ± 1.4 (4–10)	8.3 ± 1.9 (4–10)	**0.029**^**c**^
SGA	2 (8.3)	3 (11.1)	0.821
Cases of intubation	15 (62.5)	10 (37)	0.095
NCPAP use	16 (66.7)	21 (77.8)	0.531
Diuretics	7 (29.2)	4 (14.8)	0.31
Surfactant treatment	17 (70.8)	10 (37)	**0.025**^**a**^
PPHN	8 (33.3)	3 (11.1)	0.088
NO use	6 (25)	1 (3.7)	**0.042**^**a**^
BSI	4 (16.7)	6 (22.2)	0.731
Blood products	13 (54.2)	11 (40.7)	0.406
**Steroid treatment**			
Inhalation	4	2	0.402
Systemic	0	1	
Pneumothorax	2 (8.3)	3 (11.1)	1
Pulmonary hemorrhage	1 (4.2)	1 (3.7)	1
CLABSI cases	2 (8.3)	3 (11.1)	1
**Hemodynamically significant PDA**			
Medication treated	2 (8.3)	2 (7.4)	0.794
Surgery treated	1 (4.2)	0	
**Major morbidities**			
BPD	10 (41.7)	7 (25.9)	0.254
IVH grade 3–4	4 (16.7)	1 (3.7)	0.175
NEC	1 (4.2)	0	–
ROP	1 (4.2)	2 (7.4)	1
Hearing loss*	4 (23.5)	2 (8.7)	0.373
Survived to Hospital discharge	19 (79.2)	24 (88.9)	0.451

*GA* gestational age, *GW* gestational week, *SGA* small for gestational age, *NCPAP* nasal continuous positive airway pressure, *BDP* bronchopulmonary dysplasia, *IVH* intraventricular hemorrhage, *PPHN* persistent pulmonary hypertension of the newborn, *NO* nitric oxide, *NEC* necrotizing enterocolitis, *BSI* blood stream infection, *ROP* Retinopathy of prematurity, *CLABSI* Central Line Associated Bloodstream Infections, *PDA* Patent ductus arteriosus, *NA* not assessed.

^a^Fisher’s Exact Test, followed by Phi Coefficient calculation.

^b^t-test.

^c^Mann–Whitney test.

*10 missing value.

Significant values are in bold.

The neonates who did not survive in group 1 had a lower GW at PPROM (20 ± 2.3 vs 22 ± 1.2, p = 0.036) and were more susceptible to infection (40% of CLABSI cases, p = 0.036) than those who survived (Table 3). In group 2, the neonates who did not survive had a significantly lower Apgar score at 5 min than those who did survive (6 ± 2 vs 8.6 ± 1.7, respectively, p = 0.036), suffered from more PPHTN and pneumothorax (66.7% vs 4.2%, respectively, p = 0.025), and had a higher rate of CLABSI infection (66.7% vs 4.2%, respectively, p = 0.025) (Table 3).

**Table 3:** Neonatal characteristics by survival outcome.

	Group 1 (17–23 weeks)		p value	Group 2 (24–27 weeks)		p value
	Survived to discharged N = 19	Death N = 5		Survived to discharged N = 24	Death N = 3	
**Maternal characteristics**						
GW at PPROM (median, week)	22 ± 1.2 (19–23)	21 ± 2.3 (17–22)	**0.036**	26 ± 1.2 (24–27)	24 ± 1.2 (24–26)	0.139
Latency period (median, day)	50 ± 27.9 (12–105)	40 ± 31.9 (16–85)	1	17 ± 15.6 (7–61)	24 ± 5.6 (17–28)	0.635
**Neonatal outcomes**						
GA at delivery (week)	29 ± 3.3 (24–34)	26.8 ± 2.8 (23–30)	0.235	29.4 ± 2.5 (25–34)	28 ± 1.7 (27–30)	0.437
Gender (male)	10 (52.6)	4 (80)	0.358	17 (70.8)	3 (100)	0.545
Birth weight (g)	1330.6 ± 473.9 (520–2120)	1042.6 ± 434.5 (488–1560)	0.265	1393.2 ± 412 (730–2270)	983 ± 107.2 (880–1094)	0.101
Apgar 1	5.4 ± 2.5 (1–9)	4 ± 2.1 (1–7)	0.235	6.5 ± 2.7 (1–9)	4 ± 2.6 (2–7)	0.139
Apgar 5	7.5 ± 1.5 (4–10)	7.8 ± 0.8 (7–9)	0.731	8.6 ± 1.7 (4–10)	6 ± 2 (4–8)	**0.036**
SGA	1 (5.3)	1 (20)	0.380	2 (8.3)	1 (33.3)	0.308
Cases of intubation	10 (52.6)	5 (100)	0.118	7 (29.2)	3 (100)	**0.041**
NCPAP use	16 (84.2)	0	**0.001**	20 (83.3)	1 (33.3)	0.115
Surfactant treatment	12 (63.2)	5 (100)	0.272	8 (33.3)	2 (66.7)	0.535
PPHTN	5 (26.3)	3 (60)	0.289	1 (4.2)	2 (66.7)	**0.025**
NO	4 (21.1)	2 (40)	0.568	0	1 (33.3)	0.111
Blood products	9 (47.4)	4 (80)	0.327	10 (41.7)	1 (33.3)	1
Steroid treatment	4 (21.1)	0	1	2 (8.3)	1 (33.3)	0.308
Pneumothorax	1 (5.3)	1 (20)	0.380	1 (4.2)	2 (66.7)	**0.025**
BSI	2 (10.5)	2 (0.4)	0.179	4 (16.7)	2 (66.7)	0.115
CLABSI	0	2 (40)	**0.036**	1 (4.2)	2 (66.7)	**0.025**
Hemodynamically significant PDA	3 (15.8)	0	1	2 (8.3)	0	1
**Major morbidities**						
BPD	10 (52.6)	0	**0.053**	6 (25)	1 (33.3)	1
IVH grade 3–4	2 (10.5)	2 (20)	0.179	0	1 (33.3)	0.111
NEC	1 (5.3)	0	1	0	0	1
ROP	1 (5.3)	0	1	2 (8.3)	0	1
Hearing impairment*	4 (21.1)	–	NA	2 (8.3)	–	NA

Data are expressed as mean ± standard deviation, median ± standard deviation or n (%).

*GA* gestational age, *GW* gestational week, *SGA* small for gestational age, *NCPAP* nasal continuous positive airway pressure, *BDP* bronchopulmonary dysplasia, *IVH* intraventricular hemorrhage, *PPHN* persistent pulmonary hypertension of the newborn, *NO* nitric oxide, *NEC* necrotizing enterocolitis, *BSI* blood stream infection, *ROP* Retinopathy of prematurity, *CLABSI* Central Line Associated Bloodstream Infections, *PDA* Patent ductus arteriosus, *NA* not assessed.

*7 missing values in group 1 and 4 missing values in group 2.

Significant values are in bold.

**Figure 2:**
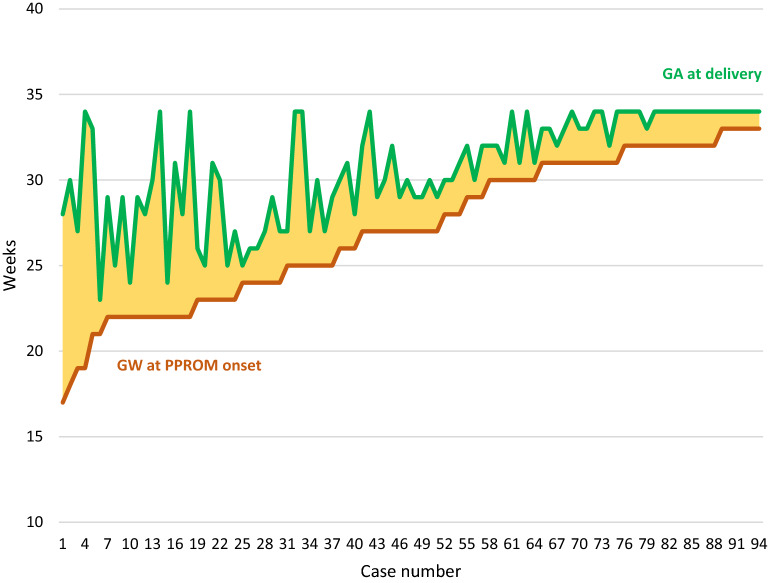
Gestational Age (GA) at delivery by gestation week (GW) at PPROM.

Spearman’s correlation was computed to assess the relationship between the following variables, GW at PPROM, GA, Apgar score at 1 and 5 min, latency period length and BW within groups 1 and 2. There were strong positive correlations between latency period and BW (r_s_ = 0.662, n = 51, p < 0.001), latency period and GA (r_s_ = 0.704, n = 51, p < 0.001) and between GA and BW (r_s_ = 0.812, n = 51, p < 0.001). Additionally, there were moderate positive correlations between GA and Apgar score 1 min (r_s_ = 0.458, n = 51, p = 0.001), BW and Apgar score 5 min (r_s_ = 0.465, n = 51, p = 0.001) and between Apgar score 1 min and 5 min (r_s_ = 0.52, n = 51, p < 0.001). Finally, the latency period length was inversely related to GW at PPROM onset (r_s_ = − 0.457, n = 51, p = 0.001) (Fig. 2). In binary logistic regression analysis, none of these variables (GA at delivery, GW at onset of PPROM, BW, Apgar score 1 and 5 min, and latency period length) remained significant in predicting survival to discharge within groups 1 and 2.

Per two-sided Fisher exact test, the rate of neonates affected by major morbidities, including ROP, IVH grade 3–4, NEC stage 2–3, BPD and hearing impairment, were similar between groups 1 and 2 (Table 2). Among the 19 surviving neonates in group 1, 63.2%^12^ were affected by one, two or three major morbidities (37%, 21% and 5.3% respectively) (Table 3) and none of the discharged neonates were affected by more than three major morbidities. In group 2, 33.3%^8^ of the surviving neonates were affected by one or two major morbidities (25% and 8.3%, respectively) (Table 3). None of the neonates in group 2 were affected by three or more major morbidities.

However, it worth noting that the prevalence of illness with irreversible damages (ROP, IVH 3–4 and hearing impairment) among the surviving neonates was only 31.6% (affected by one or two illnesses) in group 1 and only half of it, 16.7%, (affected by one illness) in group 2. None of the neonates in none of the groups was affected by all three major morbidities.

## Discussion

We found that the survival rate to discharge in neonates born to prolonged PPROM-complicated pregnancy at GW < 24 is 79.2%, a definitively non-negligible number. These neonates did not present with an increased rate of major morbidities compared to neonates born to PPROM-complicated pregnancy at GW 24 to 27.

Though advances in perinatal and neonatal practices have led to improved neonatal survival rates, they remain widely variable throughout the literature^14,15^. Our results differ from other studies that reported ranges of survival rates at periviable PPROM from as low as 24% to as high as 70%^3,16–18^. Sim et al. showed an overall neonatal survival rate to discharge of 33.8%, and stratification of patients into early (12 to 19 + 6 weeks of gestation) and late pre-viable PPROM (20 to 23 + 6 weeks of gestation) revealed a 3.6-fold increase in survival rate in the latter group (12.2% versus 43.8%, p < 0.001)^16^. Additionally, the reported neonatal survival rate to discharge was 20% in the retrospective study done by Linehan et al. in PPROM diagnosed between 14 and 23 + 6 weeks of gestation^17^. Esteves et al. found that neonatal survival rate to discharge of PPROM at GW between 18 and 20 was 18.7% and between 22 and 24 was 42.8%^18^.

Another finding from our study was that 37.7% of the neonates born following PPROM below GW 24 and survived to discharge were not affected with major morbidities (i.e. ROP, IVH grade 3–4, NEC, BPD and hearing impairment). This finding is in the range of previously reported rates of 17.8%^18^ and 55%^19^ neonatal survival without major morbidities in a similar population. Alternatively, when only ROP, IVH stage 3–4 and hearing impairment where taken into account as irreversible morbidities, the rate of discharged neonates without those major morbidities was almost twice higher than reported.

It is worth noting that most of the mothers hospitalized in our center are orthodox religious Jews who tend to reject gynecological guidelines and refuse terminating the pregnancy as it conflicts with their personal values. Hence, these mothers benefited from expectant management of their PPROM that led in many cases to life birth. Neonate survival depends on both the GW at which membrane rupture occurs and at what GA the baby is born^5^. The same was true in our study as the infants who did not survive were from a lower GW at the time of PPROM.

We speculate that the high survival rate in our study differ from the one reported in the literature due to several reasons. First, we specifically recruited prolonged PPROM complicated pregnancies (latency period of at least 7 days), that might have led to a subset of PPROM cases with better odds of neonatal survival in excluding fetal distress or emergent medical indications. Secondly, the prolonged latency period allowed for medication therapy management that included administration of two courses of corticosteroids, antibiotics and magnesium, with the ultimate goal of reaching advanced gestational age at delivery. Indeed, 50% of the mother’s in group 1 had a latency period of at least 7 weeks.

Although several studies have shown that prolonged PPROM is associated with an increased risk of infant death and morbidity^1,3,14,16,18^, we could not see an increase in the rate of major morbidity in group 1 compared with group 2. Conversely, other studies have found that prolonged PPROM did not worsen neonatal outcomes^20^ and was associated with a decreased risk of neonatal sepsis^21^. In addition, along with higher GW at rupture, increased length of latency period increased the probability of neonatal survival^22^.

Finally, we reviewed cases of PPROM from 2014 until 2019, a more recent period than the ones indicated in previously published studies on cohorts recruited until 2015^3,15,17,19^. Advances in neonatal care might have enhanced survival rates and reduced major morbidities.

### Strengths and limitations

There are few limitations to our study: the retrospective nature of the study and secondly, the fact that the study was probably not powered enough to detect statistically significant differences, between group 1 and 2, for any of the major morbidities. On the other hand, the strength of this study is its relatively large sample of singletons born preterm after a prolonged PPROM started at gestational week 17 as data on outcomes of neonates born following prolonged early preterm premature rupture of membranes are limited.

## Conclusion

The survival rate to discharge was 79.2% in neonates born to women who had prolonged PPROM before 24 weeks of at least 7 days of latency. About one third of these infants were discharged alive without major morbidities. Our findings provide new insights to physicians when counseling women with prolonged PPROM at periviable GA.

## Data Availability

The data that support the findings of this study are available on request from the corresponding author.
